# Complete Genome Sequence of the Novel Leech Symbiont *Mucinivorans hirudinis* M3^T^

**DOI:** 10.1128/genomeA.01530-14

**Published:** 2015-02-05

**Authors:** Michael C. Nelson, Lindsey Bomar, Joerg Graf

**Affiliations:** Department of Molecular and Cell Biology, University of Connecticut, Storrs, Connecticut, USA

## Abstract

*Mucinivorans hirudinis* M3^T^ was isolated from the digestive tract of the medicinal leech, *Hirudo verbana*, and is the type species of a new genus within the *Rikenellaceae*. Here, we report the complete annotated genome sequence of this bacterium.

## GENOME ANNOUNCEMENT

Bacteria belonging to the phylum *Bacteroidetes* are commonly found within the alimentary tracts of humans and animals and have previously been shown to play numerous roles in both host development and regulation of the gut microbiome (1–5). The *Rikenellaceae* is a family within the *Bacteroidetes* for which only four genera are currently described, and few full genome sequences are available. Previous work in our lab identified and isolated a novel bacterium present in the crop of medicinal leech *Hirudo verbana* that represents a new genus and species within the *Rikenellaceae* (6, 7). Here, we report the complete annotated genome sequence of the type strain isolate for this bacterium, known as *Mucinivorans hirudinis* M3^T^ (8).

An initial Illumina mate-pair library was sequenced as 100 bp paired-ends on a single lane of a HiSeq 2000. The reads were trimmed to 36 bp and assembled using CLC Genomics Workbench (version 4.0), yielding a 2.7-Mbp draft genome consisting of 332 contigs larger than 500 bp (*N*_50_ = 21,974 bp). A preliminary analysis of the draft genome after annotation by RAST identified numerous repetitive and transposable elements and phage sequences, which hindered further assembly using available short-read methods.

Because of this complexity, Pacific Bioscience’s single-molecule real-time (SMRT) sequencing technology was chosen as an optimal sequencing strategy. Subsequently, the full genome was sequenced from two libraries, one size selected greater than 7 kb and the other not, using two SMRT cells for each library on the PacBio RS II. The genome was assembled using the Hierarchical Genome Assembly Process (HGAP) assembler (9) yielding a single contig approximately 3.15 Mbp in length, which was manually edited to circularize the overlapping ends of the genome. The final genome sequence is 3,149,227 bp and has a G+C content of 44.9%. Self dot plot analysis using Gepard (10) confirmed the presence of several large (>1.5 kbp) sequence repeats that had hindered the initial assembly.

Functional annotation by RAST combined with manual annotation and curation identified 3,015 open reading frames (ORFs), 39 tRNAs, and 2 complete ribosomal operons. As initially observed in the draft genome, more than 200 ORFs were annotated as encoding phage and/or mobile element proteins. As expected for a member of the *Bacteroidetes*, the M3^T^ genome encodes for a number of carbohydrate metabolism genes, with many of these organized in polysaccharide utilization loci (PULs) similar to those present in other *Bacteroidetes*. Other notable features of the M3^T^ genomes are a genes involved in cobalamin biosynthesis, which may be utilized to provide the leech host with vitamin B_12_ which is deficient in vertebrate blood (11, 12). More than 1,200 ORFs (~40%) were annotated as hypothetical proteins, a result not surprising for the annotation of the genome of a new genus with few annotated genomes for other genera within the *Rikenellaceae*. The availability of a new, fully sequenced and annotated genome for a novel member of the *Bacteroidetes* should allow for more thorough analysis of members of this important group of bacteria.

### Nucleotide sequence accession numbers.

Raw PacBio read data and the annotated genome were deposited in EMBL/GenBank/DDBJ under the study accession no. PRJEB5062. The annotated genome sequence is available under the accession no. HG934468.
